# Research on Algorithm of Airborne Dual-Antenna GNSS/MINS Integrated Navigation System

**DOI:** 10.3390/s23031691

**Published:** 2023-02-03

**Authors:** Ming Xia, Pengfei Sun, Lianwu Guan, Zhonghua Zhang

**Affiliations:** 1 Department of Intelligent Manufacturing and Industrial Security, Chongqing Vocational Institute of Safety & Technology, Chongqing 404020, China; 2 College of Intelligent Systems Science and Engineering, Harbin Engineering University, Harbin 150001, China

**Keywords:** GNSS/MINS integrated navigation, discrete Kalman filter, strapdown inertial navigation algorithm, error analysis, GNSS initial alignment

## Abstract

In view of the difficulties regarding that airborne navigation equipment relies on imports and the expensive domestic high-precision navigation equipment in the manufacturing field of Chinese navigable aircraft, a dual-antenna GNSS (global navigation satellite system)/MINS (micro-inertial navigation system) integrated navigation system was developed to implement high-precision and high-reliability airborne integrated navigation equipment. First, the state equation and measurement equation of the system were established based on the classical discrete Kalman filter principle. Second, according to the characteristics of the MEMS (micro-electric-mechanical system), the IMU (inertial measurement unit) is not sensitive to Earth rotation to realize self-alignment; the magnetometer, accelerometer and dual-antenna GNSS are utilized for reliable attitude initial alignment. Finally, flight status identification was implemented by the different satellite data, accelerometer and gyroscope parameters of the aircraft in different states. The test results shown that the RMS (root mean square) of the pitch angle and roll angle error of the testing system are less than 0.05° and the heading angle error RMS is less than 0.15° under the indoor static condition. A UAV flight test was carried out to test the navigation effect of the equipment upon aircraft take-off, climbing, turning, cruising and other states, and to verify the effectiveness of the system algorithm.

## 1. Introduction

Integrated navigation system research is a hot spot in the fields of aviation and aerospace [1]. At present, the most widely used integrated navigation method in the aviation field is the integration of GNSS (global navigation satellite system) and INS (inertial navigation system) [2,3]. GNSS is a system capable of global positioning and time synchronization. It can provide continuous, full-time, high-precision localization services. It has now been widely used in military, aviation, navigation, automobile, agriculture, consumer electronics and numerous other fields and has become an indispensable and significant navigation mode in people’s daily lives [4]. For integration of GNSS and INS, currently, the commonly used classification standard is divided into loose integration, tight integration and deep integration according to different coupling degrees [5,6]. In the relaxed joint system, the GNSS and INS work independently and the GNSS output navigation results are then used to filter and correct the INS solution results [7]. This approach has the advantages of small system computation, high real-time performance, easy engineering implementation and high product reliability. Currently, most integrated navigation products on the market use a loose combination scheme. A loose combination, however, has its flaws. When GNSS can receive less than four satellite signals, satellite positioning fails and the loose combination does not function properly at this time. Therefore, other sensors are needed to ensure short-term navigation accuracy of the system. Currently, many loosely integrated navigation products have been applied in practical projects in China. Among them, loosely integrated navigation products are mostly used in military weapons and equipment, such as various missiles, Long March launch vehicles and aircraft. Xi’an Precision Measurement and Control has accumulated a great deal of technology in loosely integrated products and has successively launched several low-cost GNSS/INS integrated navigation systems, which have been widely used in vehicle navigation.

At present, the integrated navigation equipment widely used in the fields of military, commercial aircraft and shipping in China is mostly based on the products of laser gyro and fiber optic gyro. The equipment presents high precision and reliability priorities, while its price is also extremely expensive. However, due to the low cost of navigable aircraft and sensitivity to the price of airborne equipment, it is not possible to use expensive fiber optic or laser gyro integrated navigation equipment, which causes most domestic navigable aircraft manufacturers to rely on imported integrated navigation equipment based on MEMS (micro-electric-mechanical system) gyro and accelerometer. Navigation devices based on MEMS devices are tiny, light-weight, low-power consumption, low-cost and convenient in later maintenance. In addition, with in-depth research of MEMS-related technologies in countries around the world in recent years, accuracy and reliability of navigation devices based on MEMS have been considerably improved [8] and their performance could meet the requirements of integrated navigation devices for navigable aircraft. However, there is still a gap in popularity and reliability compared to foreign products [9,10].

In this paper, we describe the details of the mathematical model and algorithm design of the dual-antenna GNSS/MINS integrated navigation system. First, the coordinate system commonly used in navigation systems is described and the Euler angle representation method and variation range of vehicular attitude are explained. Then, the mathematical model of the dual-antenna integrated navigation system is proposed, and the core algorithms of attitude, velocity and position update are analyzed and the error equation is derived. After that, the initial attitude alignment via accelerometer and magnetometer and pitch and heading angle alignment using dual-antenna GNSS are studied. Moreover, in the case of satellite failure, the flight state of the aircraft is judged by accelerometer and gyroscope measurements, and then the attitude angle is corrected by the accelerometer in the low dynamic flight state of the aircraft.

## 2. Introduction to the Main Index Requirements and Reference Coordinate System for Integrated Navigation Systems

Navigational equipment on a navigable aircraft provides high precision and stable attitude, position, velocity and additional information during normal flight of the aircraft to assist the aircraft system in attitude control, enabling it to move along the planned route and avoid track deviations or intersections with other aircraft track routes.

In accordance with the national aerospace standards SAE AS 8013A-2008 Minimum Performance Standard for Magnetic (Gyro Stabilized) Heading Instruments [11], SAE AS 396B-2008 Tilt Pitometer (Indicative Gyro Stabilized) (Gyro Horizon, Attitude Gyro) [12], DO-160F Airborne Equipment Environment and Test Conditions [13] and referring to the performance indicators of GNSS/INS integrated navigation system equipped with foreign navigation aircraft, the main index requirements of the proposed integrated navigation system are shown in Table 1.

**Figure 1:**
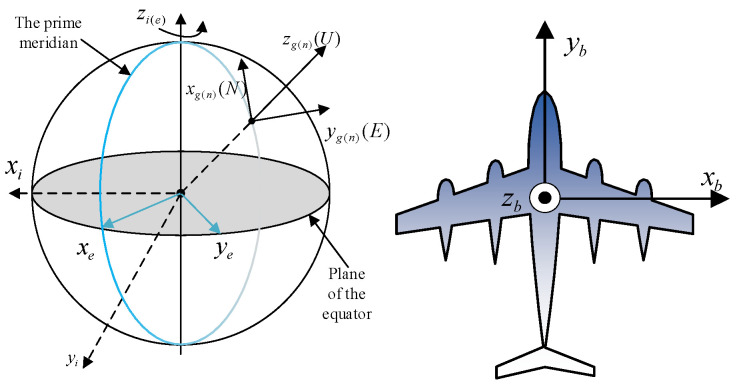
Diagram of the relationship between coordinate systems.

Integrated navigation requires a set of unified coordinate systems to accurately represent the state of the vehicle, taking different reference objects as objects under different circumstances. The common coordinate system is the inertial coordinate system (i coordinate system, $Ox_{i}y_{i}z_{i}$), Earth coordinate system (e coordinate system, $Ox_{e}y_{e}z_{e}$), geographic coordinate system (g coordinate system, $Ox_{g}y_{g}z_{g}$), navigation coordinate system (n coordinate system, $Ox_{n}y_{n}z_{n}$), vehicular coordinate system (b coordinate system, $Ox_{b}y_{b}z_{b}$) etc. [14]. The relationship between each coordinate system is shown in Figure 1.

The n system in this paper is the same as the local g system, with the x, y and z axes pointing to the northeast sky, respectively. Vehicular attitude is represented by the included angle between b series and n series; ψ is the heading angle, θ is the pitch angle and γ is the roll angle.

## 3. Establishing the Mathematical Model of Dual-Antenna GNSS/INS Integrated Navigation System

Due to the requirement of real-time performance and low cost of the system, this paper chooses the loosely integrated navigation method for data fusion. Meanwhile, in order to improve the accuracy and reliability, the indirect method is used to select the state quantity [15,16], which refers to the output navigation parameter error of the two navigation systems as the state quantity.

### 3.1. Discrete Kalman Filter

A solo navigation mode has its own limitations. Filtering and fusion of data from various sensors can make up for the disadvantages of solo navigation method and obtain a navigation system with higher accuracy and reliability. At present, the most widely used integrated navigation filtering method is Kalman and its improved algorithms [6].

First, the system state space model is provided.

(1) $$\{\begin{array}{c} X_{k}=\Phi_{k/k-1}X_{k-1}+\Gamma_{k-1}W_{k-1}\\ Z_{k}=H_{k}X_{k}+R_{k} \end{array}$$

where $X_{k}$ is the state vector at time $t_{k}$; $Z_{k}$ is the direction-finding quantity at time $t_{k}$; $\Phi_{k/k-1}$ is the state transition matrix from time $t_{k-1}$ to time $t_{k}$; $\Gamma_{k-1}$ is the noise distribution matrix; $H_{k}$ is the measurement matrix; $W_{k-1}$ is the system noise vector and $R_{k}$ is the measurement noise vector. Assuming that they are white noises subject to zero-mean Gaussian distribution, the following equation is satisfied.

(2) $$\{\begin{array}{c} E[W_{k}]=0,E[W_{k}W_{j}^{T}]=Q_{k}\delta_{kj}\\ E[V_{k}]=0,E[V_{k}V_{j}^{T}]=R_{k}\delta_{kj}\\ E[W_{k}V_{j}^{T}]=0 \end{array}$$

where $Q_{k}$ is the process noise matrix, $R_{k}$ is the measurement noise matrix, $\delta_{kj}$ is Crohn’s function, $Q_{k}$ is generally required to be non-negative definite and $R_{k}$ is positive definite, that is $Q_{k}\geq 0$ and $R_{k}>0$.

KF (Kalman filter) is mainly divided into prediction and correction. The prediction part is divided into state one-step prediction and MSE (mean square error) matrix prediction. The formula is as follows.

(3) $${\hat{X}}_{k/k-1}=\Phi_{k/k-1}{\hat{X}}_{k-1}$$

(4) $$P_{k/k-1}=\Phi_{k/k-1}P_{k-1}\Phi_{k/k-1}^{T}+\Gamma_{k-1}Q_{k-1}\Gamma_{k-1}^{T}$$

In the correction step, Kalman gain is calculated first, as shown in (5).

(5) $$K_{k}=P_{k/k-1}H_{k}^{T}{\left(H_{k}P_{k/k-1}H_{k}^{T}+R\right)}^{-1}$$

Then, state estimation and MSE matrix estimation are performed, as shown in (6) and (7).

(6) $${\hat{X}}_{k}={\hat{X}}_{k/k-1}+K_{k}(Z_{k}-H_{k}{\hat{X}}_{k/k-1})$$

(7) $$P_{k}=(I-K_{k}H_{k})P_{k/k-1}$$

### 3.2. State Equation, Measurement Equation and Parameter Selection of Design Navigation System

The state equation and measurement equation of the system are used to reflect the state characteristics of the system and the relationship between measurement information and state, respectively. The simplified navigation system equation of state and measurement equation are designed separately, and the parameter selection method for the system equation of state and measurement equation is introduced.

The equation of state of the integrated navigation system is as follows.

(8) $$\dot{X}{\left(t\right)}_{15\times 1}=F{\left(t\right)}_{15\times 15}X{\left(t\right)}_{15\times 1}+G{\left(t\right)}_{15\times 12}W{\left(t\right)}_{12\times 1}$$

where F(t) is the state transition matrix of the system; X(t) is the state vector matrix; G(t) is the system noise distribution matrix; W(t) is the system noise matrix.

This paper selects MINS ‘attitude misalignment angle ${\left[\begin{array}{ccc} \phi_{E} & \phi_{N} & \phi_{U} \end{array}\right]}^{T}$, northeast sky velocity error ${\left[\begin{array}{ccc} \delta v_{E} & \delta v_{N} & \delta v_{U} \end{array}\right]}^{T}$, longitude and latitude height position error ${\left[\begin{array}{ccc} \delta L & \delta \lambda & \delta h \end{array}\right]}^{T}$, gyroscope correlation drift ${\left[\begin{array}{ccc} \varepsilon_{gx}^{b} & \varepsilon_{gy}^{b} & \varepsilon_{gz}^{b} \end{array}\right]}^{T}$ and accelerometer correlation drift ${\left[\begin{array}{ccc} \nabla_{ax}^{b} & \nabla_{ay}^{b} & \nabla_{az}^{b} \end{array}\right]}^{T}$ as state vector (15 dimensions in total), as follows.

(9) $$X\left(t\right)=[\phi_{E}\;\phi_{N}\;\phi_{U}\;\delta v_{E}\;\delta v_{N}\;\delta v_{U}\;\delta L\;\delta \lambda \;\delta h\;\varepsilon_{gy}^{b}\;\varepsilon_{gz}^{b}\;\nabla_{ax}^{b}\;\nabla_{ay}^{b}\;\nabla_{az}^{b}]$$

The state transition matrix of the system at epoch *t* is as follows.

(10) $$F\left(t\right)={\left[\begin{array}{cc} F_{I}{\left(t\right)}_{9\times 9} & F_{C}{\left(t\right)}_{9\times 6}\\ 0_{6\times 9} & F_{\varepsilon }{\left(t\right)}_{6\times 6} \end{array}\right]}_{15\times 15}$$

where $F_{I}\left(t\right)$ is the error matrix of the strapdown inertial navigation system, which can be expressed in the following form.

(11) $$F_{I}\left(t\right)={\left[\begin{array}{ccc} 0_{3\times 3} & 0_{3\times 3} & 0_{3\times 3}\\ f_{sf}^{n}\times & 0_{3\times 3} & 0_{3\times 3}\\ 0_{3\times 3} & F_{3\times 3}^{p} & 0_{3\times 3} \end{array}\right]}_{9\times 9}$$

(12) $$F_{C}\left(t\right)={\left[\begin{array}{ll} -C_{b}^{n} & 0_{3\times 3}\\ 0_{3\times 3} & C_{b}^{n}\\ 0_{3\times 3} & 0_{3\times 3} \end{array}\right]}_{9\times 6}$$

(13) $$F_{\varepsilon }\left(t\right)={\left[\begin{array}{cc} -\alpha_{g} & 0_{3\times 3}\\ 0_{3\times 3} & -\alpha_{a} \end{array}\right]}_{6\times 6}$$

where $C_{b}^{n}$ is the ideal direction cosine matrix, $f_{sf}^{n}$ is the measurement of accelerometer in navigation frame, $\alpha_{s}=diag\left(\begin{array}{ccc} 1/\tau_{sx} & 1/\tau_{sy} & 1/\tau_{sz} \end{array}\right)\left(s=g,a\right)$ and $1/\tau_{si}\left(s=g,a;i=x,y,z\right)$ are Markov time correlation constants.

In this paper, random white noise of gyroscope is ${\left[\begin{array}{ccc} w_{g\varepsilon x} & w_{g\varepsilon y} & w_{g\varepsilon z} \end{array}\right]}^{T}$, random white noise of accelerometer is ${\left[\begin{array}{ccc} w_{a\varepsilon x} & w_{a\varepsilon y} & w_{a\varepsilon z} \end{array}\right]}^{T}$, first-order Markov drive white noise of gyroscope ${\left[\begin{array}{ccc} \eta_{gx} & \eta_{gy} & \eta_{gz} \end{array}\right]}^{T}$ and first-order Markov drive white noise of accelerometer ${\left[\begin{array}{ccc} \eta_{ax} & \eta_{ay} & \eta_{az} \end{array}\right]}^{T}$ are taken as the noise of the system, so the system noise matrix is shown in the following equation.

(14) $$W\left(t\right)=[w_{g\varepsilon x}\;w_{g\varepsilon y}\;w_{g\varepsilon z}\;w_{a\varepsilon x}\;w_{a\varepsilon y}\;w_{a\varepsilon z}\;\eta_{gx}\;\eta_{gy}\;\eta_{gz}\;\eta_{ax}\;\eta_{ay}\;\eta_{az}]$$

The covariance matrix corresponding to the noise matrix P is shown in the following equation.

(15) $$P\left(t\right)=diag[\sigma_{gx}^{2}\;\sigma_{gy}^{2}\;\sigma_{gz}^{2}\;\sigma_{ax}^{2}\;\sigma_{ay}^{2}\;\sigma_{az}^{2}\;2\alpha \sigma_{g\varepsilon x}^{2}\;2\alpha \sigma_{g\varepsilon y}^{2}\;2\alpha \sigma_{g\varepsilon z}^{2}\;2\beta \sigma_{a\varepsilon x}^{2}\;2\beta \sigma_{a\varepsilon y}^{2}\;2\beta \sigma_{a\varepsilon z}^{2}]$$

The system noise distribution matrix is shown in Equation (16) below.

(16) $$G\left(t\right)={\left[\begin{array}{llll} -C_{b}^{n} & 0_{3\times 3} & 0_{3\times 3} & 0_{3\times 3}\\ 0_{3\times 3} & C_{b}^{n} & 0_{3\times 3} & 0_{3\times 3}\\ 0_{3\times 3} & 0_{3\times 3} & 0_{3\times 3} & 0_{3\times 3}\\ 0_{3\times 3} & 0_{3\times 3} & I_{3\times 3} & 0_{3\times 3}\\ 0_{3\times 3} & 0_{3\times 3} & 0_{3\times 3} & I_{3\times 3} \end{array}\right]}_{15\times 12}$$

The designed dual-antenna GNSS/MINS loosely integrated navigation system uses nine dimensions of position, velocity and attitude data as observed measurement. The measurement equation is shown as follows.

(17) $$Z{\left(t\right)}_{9\times 1}=H{\left(t\right)}_{9\times 15}X{\left(t\right)}_{15\times 1}+R{\left(t\right)}_{9\times 1}$$

where $Z\left(t\right)={\left[\begin{array}{ccc} {\vec{\Phi }}_{I}^{n}-{\vec{\Phi }}_{G/M}^{n} & {\vec{v}}_{I}^{n}-{\vec{v}}_{G}^{n} & {\vec{p}}_{I}^{n}-{\vec{p}}_{G}^{n} \end{array}\right]}^{T}$,$\Phi_{I}^{n}$ are the attitude calculated by INS and $\Phi_{G/M}^{n}$ is the attitude angle provided by GNSS and magnetometer. Since GNSS can only provide heading and pitch angle, when the system is carrying out flight attitude recognition and confirming that the current state can use the accelerometer for error compensation, it will use the accelerometer to obtain the roll angle measurement value; otherwise, the roll angle measurement error will be set to zero. $v_{I}^{n}$ and $v_{G}^{n}$ are the velocity output information of INS and GNSS, and $p_{I}^{n}$ and $p_{G}^{n}$ are the latitude, longitude and height output information of INS and GNSS, respectively.

In $H\left(t\right)={\left[\begin{array}{ccc} H_{\Phi }{\left(t\right)}_{3\times 15} & H_{v}{\left(t\right)}_{3\times 15} & H_{p}{\left(t\right)}_{3\times 15} \end{array}\right]}^{T}$.

(18) $$H_{\Phi }=\left[\begin{array}{cc} I_{3\times 3} & 0_{3\times 12} \end{array}\right]$$

(19) $$H_{v}=\left[\begin{array}{ccc} 0_{3\times 3} & I_{3\times 3} & 0_{3\times 9} \end{array}\right]$$

(20) $$H_{p}=\left[\begin{array}{ccc} 0_{3\times 6} & I_{3\times 3} & 0_{3\times 6} \end{array}\right]$$

The measurement noise matrix is $R\left(t\right)={\left[\begin{array}{ccc} R_{\Phi }\left(t\right) & R_{v}\left(t\right) & R_{p}\left(t\right) \end{array}\right]}^{T}$, where $R_{\Phi }\left(t\right)$ is the white noise of GNSS and magnetometer, $R_{v}\left(t\right)$ is the white noise of GNSS receiver velocity and $R_{p}\left(t\right)$ is position measurement white noise.

Under the condition of the satellite signal being effective, it is preferred to use the heading and pitch signals of the dual-antenna GNSS as the heading reference information and the MINS heading calculation difference as the measurement value. Magnetometer data validity judgments are completed when the dual-antenna GNSS signal is not valid or when the accuracy factor level does not meet the requirements. When its validity meets the requirements, it is taken as the head reference information, the MINS solution heading difference is taken as the measurement value and the pitch error information is taken as 0. When neither of them meet the requirements, the integrated filtering of attitude angle will not be performed.

## 4. Calculation Error of Dual-Antenna GNSS System and Design of Updating Algorithm for Strapdown Inertial Navigation System

### 4.1. Dual-Antenna GNSS System and Precision Factor

GNSS consists of space satellite, ground console and user receiving terminal. GNSS space satellite systems include GPS (Global Positioning System), GLONASS, GALILEO, Beidou, etc. Each system consists of several satellites in different orbits. Each satellite continuously sends signals to Earth to help complete navigation and positioning, timing and short message communication and other functions. From the perspective of positioning methods, GNSS can be divided into absolute positioning and differential positioning. Based on single-antenna GNSS, with antenna 1 as the reference station and antenna 2 as the terminal station, the coordinates of the two antennas are obtained by differential positioning method, and the baseline vector between the two antennas is solved so as to complete the heading and pitch angle measurement.

The calculation error of GNSS can be judged by three precision factors, among which PDOP (position dilution of precision) represents the square and square root of standard deviation between longitude, latitude and height, VDOP (vertical dilution of precision) represents the square and square root of standard deviation between longitude and latitude and HDOP (horizontal dilution of precision) represents the standard deviation of elevation [17].The relationship between them can be expressed as $PDOP^{2}=HDOP^{2}+VDOP^{2}$ [18]. By discriminating the value of the three accuracy factors, the quality of the current satellite calculation can be identified. The better the quality of the solution, the smaller the error and the corresponding precision factor value will be. Combine with other methods to compensate.

### 4.2. Updating Algorithm of Strapdown Inertial Navigation System

The strapdown inertial navigation system is firmly connected to the carrier, and the angular velocity and specific force of the carrier are measured by collecting the data of the internal inertial measurement unit. Then, the attitude, velocity and position information of the carrier are calculated by the inertial navigation updating equation. MINS uses MEMS gyro and MEMS accelerometer devices, making it smaller and cheaper than other inertial navigation systems and allowing it to play a more important role in more areas.

**Figure 2:**
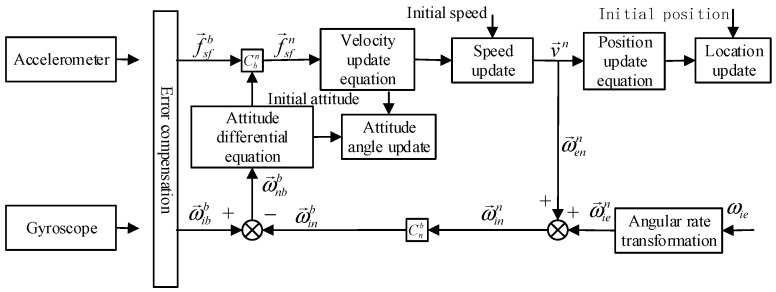
Schematic diagram of inertial navigation solution.

The MINS obtains carrier-specific force information from the MEMS accelerometer inside the IMU, and the MEMS gyroscope obtains carrier angular rate information. After error compensation, coordinate system change, updating equation computation and other steps, the latest navigation information of the carrier is obtained. The inertial navigation solution graph is shown in Figure 2 below.

First, the original carrier-specific force information obtained by the accelerometer is compensated for error to eliminate the accelerometer bias and installation error, and then the vector value ${\vec{f}}_{sf}^{n}$ of the specific force in the navigation coordinate system is obtained by multiplying the compensated specific force vector ${\vec{f}}_{sf}^{b}$ by the directional cosine matrix $C_{b}^{n}$.

Next, the velocity vector ${\vec{v}}^{n}$ under the navigation coordinate system is obtained by substituting ${\vec{f}}_{sf}^{n}$ into the velocity update equation, and then the position coordinate quantity under the updated navigation coordinate system is obtained by substituting ${\vec{v}}^{n}$ into the position update equation.

The angular rate ${\vec{\omega }}_{ib}^{b}$ is obtained from the gyro data after the effects of its bias and mounting errors are removed by error compensation. Due to the motion of the carrier, the carrier will generate an angular rate ${\vec{\omega }}_{en}^{n}$ with respect to the e coordinate system under the n coordinate system, which is given by:

(21) $${\vec{\omega }}_{en}^{n}={\left[-\frac{v_{N}}{R_{M}+h}\frac{v_{E}}{R_{N}+h}\frac{v_{E}}{R_{N}+h}\tan L\right]}^{T}$$

where $R_{M}$ is the radius of curvature of the meridian circle, $R_{N}$ is the radius of curvature of the prime unitary circle, h is the altitude and L is the local latitude. When the curvature of Earth is neglected as a sphere, it can be approximated as $R_{M}=R_{N}=\bar{R}=6,371,001\;m$.

The rotation rate of Earth is $\omega_{ie}$. Using Equation ${\vec{\omega }}_{ie}^{n}={\left[0\;\omega_{ie}\cos L\;\omega_{ie}\sin L\right]}^{T}$, we obtain its projection under the n coordinate system. Add ${\vec{\omega }}_{en}^{n}$ and ${\vec{\omega }}_{ie}^{n}$ to obtain the rotation angular rate ${\vec{\omega }}_{in}^{n}$ of the n coordinate system with respect to the I-system, and then transform the orientation cosine matrix $C_{n}^{b}$ to obtain the angular rate projection ${\vec{\omega }}_{in}^{b}$ of the B-system.

Finally, the angular rate projection ${\vec{\omega }}_{nb}^{b}$ of the B-system with respect to the N-system in the B-system is obtained by subtracting ${\vec{\omega }}_{in}^{b}$ from ${\vec{\omega }}_{ib}^{b}$ and substituting it into the attitude differential equation ${\dot{C}}_{b}^{n}=C_{b}^{n}({\vec{\omega }}_{nb}^{b}\times )$ to update the equivalent cosine matrix and the attitude angle.

Due to the large noise of the MEMS gyroscope, it is difficult to perceive the angular rate variations of the system with respect to the I-system caused by the rotation of Earth and the motion of the aircraft. To simplify the calculation, the angular rate ${\vec{\omega }}_{in}^{b}$ can be taken to be zero.

Inertial navigation update algorithm includes attitude update algorithm, velocity update algorithm and position update algorithm, among which accuracy of attitude update algorithm has a decisive influence on accuracy of the whole inertial navigation system.

(1) Attitude updating algorithm

There are three common ways to represent the attitude, which are Euler angle, direction cosine matrix and Quaternion. Euler angle is the most intuitive representation, which directly reflects the current vehicular attitude through the size of heading angle, pitch angle and roll angle. However, when the pitch angle is close to 90°, the Euler angle will be used to calculate the singular value, resulting in the phenomenon of universal joint deadlock [19], and it is impossible to measure the full attitude. The direction cosine matrix has 9 differential equations, which requires a large amount of computation and is not conducive to the real-time performance of navigation computer. Quaternion has been widely used because it has only four elements, a small amount of computation and can overcome the influence of singular value on attitude measurement. In this paper, Quaternion will be used for attitude representation, and its expression method is as follows.

(22) $$Q=q_{0}+q_{1}\vec{i}+q_{2}\vec{j}+q_{3}\vec{k}$$

Quaternion is used to represent the relative conversion relationship between the vehicular system and the navigation system, and the Quaternion expression of the direction cosine matrix can be obtained in Equation (23).

(23) $$C_{b}^{n}=\left[\begin{array}{ccc} q_{0}^{2}+q_{1}^{2}-q_{2}^{2}-q_{3}^{2} & 2(q_{1}q_{1}-q_{0}q_{3}) & 2(q_{1}q_{3}+q_{0}q_{2})\\ 2(q_{1}q_{2}+q_{0}q_{3}) & 1-(q_{1}^{2}+q_{3}^{2}) & 2(q_{2}q_{3}-q_{0}q_{1})\\ 2(q_{1}q_{3}-q_{0}q_{2}) & 2(q_{2}q_{3}+q_{0}q_{1}) & q_{0}^{2}-q_{1}^{2}-q_{2}^{2}+q_{3}^{2} \end{array}\right]$$

Using Euler angles to represent the direction cosine matrix is shown below.

(24) $$C_{b}^{n}=\left[\begin{array}{ccc} \cos\gamma \cos\psi +\sin\theta \sin\gamma \sin\psi & \cos\theta \sin\psi & \sin\gamma \cos\psi -\sin\theta \cos\gamma \sin\psi \\ -\cos\gamma \sin\psi +\sin\theta \sin\gamma \cos\psi & \cos\theta \cos\psi & -\sin\gamma \sin\psi -\sin\theta \cos\gamma \cos\psi \\ -\cos\theta \sin\gamma & \sin\theta & \cos\gamma \cos\theta \end{array}\right]$$

Euler’s angle is expressed as a Quaternion.

(25) $$\left\{ \begin{array}{l} \theta =\arcsin(2q_{2}q_{3}+2q_{0}q_{1})\\ \gamma =-\arctan((2q_{1}q_{3}-2q_{0}q_{2})/(q_{0}^{2}-q_{1}^{2}-q_{2}^{2}+q_{3}^{2}))\\ \psi =\arctan((2q_{1}q_{2}-2q_{0}q_{3})/(q_{0}^{2}-q_{1}^{2}+q_{2}^{2}-q_{3}^{2})) \end{array}\right.$$

The Quaternion differential equation is as follows.

(26) $$\left[\begin{array}{c} {\dot{q}}_{0}\\ {\dot{q}}_{1}\\ {\dot{q}}_{2}\\ {\dot{q}}_{3} \end{array}\right]=\frac{1}{2}\left[\begin{array}{cccc} 0 & -\omega_{x}^{b} & -\omega_{y}^{b} & -\omega_{z}^{b}\\ \omega_{x}^{b} & 0 & \omega_{z}^{b} & -\omega_{y}^{b}\\ \omega_{y}^{b} & -\omega_{z}^{b} & 0 & \omega_{x}^{b}\\ \omega_{z}^{b} & \omega_{y}^{b} & -\omega_{x}^{b} & 0 \end{array}\right]\left[\begin{array}{c} q_{0}\\ q_{1}\\ q_{2}\\ q_{3} \end{array}\right]$$

where $\omega_{x}^{b},\;\omega_{y}^{b},\;\omega_{z}^{b}$ is the angular velocity of the vehicular around the x, y and z axes of the vehicular coordinate system.

When non-fixed axis rotation occurs, direct Euler angle solution will introduce rotational non-exchangeable error to affect the accuracy of solution. Therefore, an equivalent rotation vector should be introduced to reduce the impact of error on the accuracy. The differential equation of the Equivalent rotation vector is shown in Equation (27).

(27) $${\dot{\vec{\phi }}}_{t}={\vec{\omega }}_{t}^{b}+\frac{1}{2}\Delta {\vec{\theta }}_{t}^{b}\times {\vec{\omega }}_{t}^{b}$$

In order to ensure the accuracy and real-time performance of the solution, this paper adopts the algorithm of “monad sample + previous period” to calculate the equivalent rotation vector, and its formula is shown in Equation (28).

(28) $${\vec{\phi }}_{t}=\Delta {\vec{\theta }}_{1}^{b}+\frac{1}{12}\Delta {\vec{\theta }}_{0}^{b}\times \Delta {\vec{\theta }}_{1}^{b}$$

where the angle increment at the previous time is $\Delta {\vec{\theta }}_{0}^{b}=\int_{-T}^{0}{\vec{\omega }}_{t}^{b}dt$ and the angle increment at the current time is $\Delta {\vec{\theta }}_{1}^{b}=\int_{0}^{T}{\vec{\omega }}_{t}^{b}dt$.

By solving the above formula, the Quaternion recursive calculation formula is obtained.

(29) $$Q_{b(t)}^{n}=Q_{b(t-1)}^{n}\circ Q_{b(t)}^{b(t-1)}$$

(30) $$Q_{b(t)}^{b(t-1)}=\left[\begin{array}{c} \cos\frac{\Delta \phi_{t}}{2}\\ \frac{\Delta {\vec{\phi }}_{t}}{\Delta \phi_{t}}\sin\frac{\Delta \phi_{t}}{2} \end{array}\right]$$

where $Q_{b(t)}^{n}$ is the updated Quaternion at time t and $Q_{b(t)}^{b(t-1)}$ is the change value of the Quaternion at time t−1 to t.

(1) Velocity update algorithm

To calculate Earth’s rotation velocity $\omega_{ie}^{e}\approx 0.025{}^{\circ}/s$, assume that the cruising velocity of a navigable aircraft is v=230 km/h and its altitude is h=2000 m; its maximum angular velocity of n series relative to e series is $v/(\bar{R}+h)\approx 0.036\;{}^{\circ}/h$, both of which will be submerged in the noise of MEMS gyroscope. Therefore, the simplified inertial navigation specific force equation is directly used in this paper.

(31) $${\dot{\vec{v}}}_{en}^{n}=C_{b}^{n}{\vec{f}}_{sf}^{b}+{\vec{g}}^{n}$$

The velocity renewal equation is as follows.

(32) $${\vec{v}}_{t}^{n}={\vec{v}}_{t-1}^{n}+C_{b(t-1)}^{n(t-1)}(\Delta {\vec{v}}_{t}^{b}+\frac{1}{2}\Delta {\vec{\theta }}_{t}^{b}\times \Delta {\vec{v}}_{t}^{b})+g^{n}T$$

where ${\vec{v}}_{k}^{n}$ is the velocity of the vehicular under the n system at time $t_{k}$, $\Delta {\vec{v}}_{k}^{b}$ is the specific force increment output by the accelerometer during the period $t_{k-1}$ to $t_{k}$, ${\vec{\theta }}_{k}^{b}$ is the angular velocity increment of the vehicular during the period $t_{k-1}$ to $t_{k}$ and T is the sampling period.

(2) Location update algorithm

Differential equations with updated positions are as follows.

(33) $$\left[\begin{array}{c} \dot{L}\\ \dot{\lambda }\\ \dot{h} \end{array}\right]=\left[\begin{array}{lll} 0 & \frac{1}{R_{M}+h} & 0\\ \frac{\sec L}{R_{N}+h} & 0 & 0\\ 0 & 0 & 1 \end{array}\right]\left[\begin{array}{c} v^{E}\\ v^{N}\\ v^{U} \end{array}\right]$$

### 4.3. Error Analysis of Strapdown Inertial Navigation System and Establishment of Error Equation and Model

The updating equations of the Jet-Link inertial guidance system are defaulted to operate the system without errors under ideal conditions. However, in actual application, measurement and operation of the inertial devices can lead to errors, which are further accumulated by the navigation algorithm, and the overall accuracy of the system will gradually decrease. For this reason, error analysis and modeling of the system are needed to reduce the impact of errors.

(1) Attitude error Equation

Under the assumption that there is no error in the system, the ideal direction cosine matrix is $C_{b}^{n}$. However, realistic rotation will have errors added to it, resulting in the actual calculated direction cosine matrix being $C_{b}^{n^{\prime }}$. That means that there is a deviation between the calculated navigation coordinate system $n^{\prime }$ and the ideal navigation coordinate system n obtained from the calculation, and according to the chain multiplication rule, there is:

(34) $$C_{b}^{n^{\prime }}=C_{n}^{n^{\prime }}C_{b}^{n}$$

The equivalent rotation vector from the n system to the $n^{\prime }$ system is denoted as $\vec{\phi }$ and referred to as the misalignment angle error. With short sampling time, $\vec{\phi }$ is a very small quantity and can be approximated using the equation for the relationship between equivalent rotation vector and direction cosine [20], which is obtained as follows:

(35) $$C_{b}^{n}{}^{\prime }\approx \left[I_{3\times 3}-\left(\vec{\phi }\times \right)\right]C_{b}^{n}$$

Without considering its angular rate with respect to the inertial coordinate system caused by the motion of the navigation coordinate system, there is equation ${\dot{C}}_{b}^{n}=C_{b}^{n}\left({\vec{\omega }}_{ib}^{b}\times \right)$, according to which the differential equation for the direction cosine matrix in the presence of error is obtained as:

(36) $${\dot{C}}_{b}^{n^{\prime }}=C_{b}^{n}{}^{\prime }\left({\vec{\tilde{\omega }}}_{ib}^{b}\times \right)$$

where ${\vec{\tilde{\omega }}}_{ib}^{b}$ is the vehicular angular rate containing the error; the equation is as follows:

(37) $${\vec{\tilde{\omega }}}_{ib}^{b}={\vec{\omega }}_{ib}^{b}+{\vec{\varepsilon }}_{g}^{b}+{\vec{w}}_{g\varepsilon }$$

where ${\vec{\varepsilon }}_{g}^{b}$ is the first-order Markov process drift of the gyroscope and ${\vec{w}}_{g\varepsilon }$ is the white noise of the gyroscope.

Differentiating the two sides of Equation (35) yields:

(38) $${\dot{C}}_{b}^{n^{\prime }}=\frac{d\left[I_{3\times 3}-\left(\vec{\phi }\times \right)\right]C_{b}^{n}}{dt}=-\left(\dot{\vec{\phi }}\times \right)C_{b}^{n}+\left(I_{3\times 3}-\left(\vec{\phi }\times \right)\right){\dot{C}}_{b}^{n}$$

Associating Equations (36) and (37), the following equation can be obtained:

(39) $$-\left(\dot{\vec{\phi }}\times \right)C_{b}^{n}+\left(I_{3\times 3}-\left(\vec{\phi }\times \right)\right){\dot{C}}_{b}^{n}=C_{b}^{n}{}^{\prime }\left({\vec{\tilde{\omega }}}_{ib}^{b}\times \right)$$

Substituting Equations (35)–(37) into (39), it can be obtained:

(40) $$-\left(\dot{\vec{\phi }}\times \right)C_{b}^{n}+\left[I_{3\times 3}-\left(\vec{\phi }\times \right)\right]C_{b}^{n}\left({\vec{\omega }}_{ib}^{b}\times \right)=\left[I_{3\times 3}-\left(\vec{\phi }\times \right)\right]C_{b}^{n}\left[\left({\vec{\omega }}_{ib}^{b}+{\vec{\varepsilon }}_{g}^{b}+{\vec{\omega }}_{\varepsilon }\right)\times \right]$$

By swapping the two sides and neglecting the second-order minima in them, posture Equation (41) is obtained:

(41) $$\dot{\vec{\phi }}=-C_{b}^{n}\left({\vec{\varepsilon }}_{g}^{b}+{\vec{\omega }}_{g\varepsilon }\right)$$

(2) Velocity error Equation

As with the attitude error, in the actual navigation calculation, the inclusion of various errors due to it will result in error between the velocity ${\vec{\tilde{v}}}^{n^{\prime }}$ obtained from the navigation calculation and the ideal velocity ${\vec{v}}^{n}$. The error calculation equation is as follows:

(42) $$\delta {\vec{v}}^{n}={\vec{\tilde{v}}}^{n^{\prime }}-{\vec{v}}^{n}$$

Differentiating both sides of Equation (42), it is possible to obtain:

(43) $$\delta {\dot{\vec{v}}}^{n}={\dot{\vec{\tilde{v}}}}^{n^{\prime }}-{\dot{\vec{v}}}^{n}$$

For ease, the simplified differential equation for the inertial conductivity ratio Equation (43) is rewritten as follows.

(44) $${\dot{\vec{v}}}^{n}=C_{b}^{n}{\vec{f}}_{sf}^{b}+{\vec{g}}^{n}$$

Without considering the error of gravity, the actual calculated differential equation for the inertia ratio force is obtained as follows.

(45) $${\dot{\vec{\tilde{v}}}}^{n^{\prime }}={\tilde{C}}_{b}^{n^{\prime }}{\vec{\tilde{f}}}_{sf}^{b}+{\vec{g}}^{n}$$

where

(46) $${\vec{\tilde{f}}}_{sf}^{b}={\vec{f}}_{sf}^{b}+{\vec{\nabla }}_{a}^{b}+{\vec{w}}_{a\varepsilon }$$

where ${\vec{\nabla }}_{a}^{b}$ is the first-order Markov process drift of the accelerometer and ${\vec{w}}_{a\varepsilon }$ is the white noise of the accelerometer.

The ratio differential equation obtained by using the actual calculation is subtracted from the ideally obtained ratio differential equation. Equation (45) minus (44), the two error differential equations can be obtained as:

(47) $$\delta {\dot{\vec{v}}}^{n}={\dot{\vec{\tilde{v}}}}^{n^{\prime }}-{\dot{\vec{v}}}^{n}={\tilde{C}}_{b}^{n^{\prime }}{\vec{\tilde{f}}}_{sf}^{b}-C_{b}^{n}{\vec{f}}_{sf}^{b}$$

Then, bring Equations (35) and (46) into (47), expand and neglect the second-order minima about the error; the velocity error equation is obtained:

(48) $$\delta {\dot{\vec{v}}}^{n}=C_{b}^{n}{\vec{f}}_{sf}^{b}\times \vec{\phi }+C_{b}^{n}\left({\vec{\nabla }}_{a}^{b}+{\vec{w}}_{a\varepsilon }\right)$$

(3) Position error Equation

By deviating the equations corresponding to the differential equations of latitude, longitude and altitude of Equation (33), the following equation can be obtained.

(49) $$\{\begin{array}{c} \delta \dot{L}=\delta v_{N}/\left(R_{M}+h\right)-\delta h/{\left(R_{M}+h\right)}^{2}\\ \delta \dot{\lambda }=\delta v_{E}\cdot \sec L/\left(R_{N}+h\right)+\delta L\cdot v_{E}\cdot \sec L\cdot \tan L/\left(R_{N}+h\right)-\delta h\cdot v_{E}\cdot \sec L/{\left(R_{N}+h\right)}^{2}\\ \delta \dot{h}=\delta v_{U} \end{array}$$

Neglecting the effect of the second-order minima in it, it is written in matrix form as follows.

(50) $$\left[\begin{array}{c} \delta \dot{L}\\ \delta \dot{\lambda }\\ \delta \dot{h} \end{array}\right]=\left[\begin{array}{ccc} 0 & \frac{1}{R_{M}+h} & 0\\ \frac{\sec L}{R_{N}+h} & 0 & 0\\ 0 & 0 & 1 \end{array}\right]\left[\begin{array}{c} \delta v_{E}\\ \delta v_{N}\\ \delta v_{U} \end{array}\right]=F_{3\times 3}^{p}\left[\begin{array}{c} \delta v_{E}\\ \delta v_{N}\\ \delta v_{U} \end{array}\right]$$

(4) Mathematical model of gyroscope error

The main error of the gyroscope can be obtained according to Equation (37), which contains the first-order Markov process drift error of the gyroscope and the white noise random drift of the gyroscope, and the MSE of the white noise is [21].

The mathematical model of the first-order Markov process drift error ${\vec{\varepsilon }}_{g}^{b}$ is as follows.

(51) $${\vec{\varepsilon }}_{g}^{b}=-\alpha_{g}{\vec{\varepsilon }}_{g}^{b}+{\vec{\eta }}_{g}$$

where α is the Markov correlation time, ${\vec{\eta }}_{g}$ is the Markov correlation white noise with mean squared difference $\sigma_{g\varepsilon }$. The drift error is converted to the navigation coordinate system using equation ${\vec{\varepsilon }}_{g}^{n}=C_{b}^{n}{\vec{\varepsilon }}_{g}^{b}$.

(5) Mathematical model of accelerometer error

According to Equation (46), the main error ${\vec{e}}_{a}={\vec{\nabla }}_{a}^{b}+{\vec{\eta }}_{a}$ of the accelerometer can be obtained, which contains the first-order Markov process drift error ${\vec{\nabla }}_{a}^{b}$ of the accelerometer, the white noise random drift ${\vec{w}}_{a\varepsilon }$ of the accelerometer and the MSE ${\vec{w}}_{a\varepsilon }$ of the white noise $\sigma_{a}$.

The mathematical model of the first-order Markov process drift error ${\vec{\nabla }}_{a}^{b}$ is as follows.

(52) $${\vec{\nabla }}_{a}^{b}=-\alpha_{a}{\vec{\nabla }}_{a}+{\vec{\eta }}_{a}$$

In the same mathematical model as the gyroscope error, β in the above equation is the Markov correlation time and ${\vec{\eta }}_{a}$ is the Markov correlation white noise with mean squared deviation $\sigma_{a\varepsilon }$. The drift error is converted to the navigation coordinate system using equation ${\vec{\nabla }}_{a}^{n}=C_{b}^{n}{\vec{\nabla }}_{a}^{b}$.

### 4.4. An Initial Alignment Algorithm of Dual-Antenna GNSS and Magnetometer-Assisted MINS

Before normal operation of the Jet-Link inertial guidance system, an initial alignment is first required to determine the initial position, velocity, attitude and other information of the vehicle in the current state. Among them, initial alignment of vehicular heading is particularly important and is the difficult part of the alignment phase [22]. A high-accuracy inertial guidance system can be sensitive to the rotation of Earth and achieve the heading alignment function without other external auxiliary information. However, the MEMS IMU is noisy and insensitive and cannot measure the angular rate of Earth’s rotation; for this reason, this paper will use magnetometer and dual-antenna GNSS for initial heading angle alignment. In this paper, the initial velocity is defaulted to zero, and the positioning output of GNSS after stable operation is used as the initial position.

(1) The initial attitude alignment is based on angular velocity meter and magnetometer

The vector measured by the accelerometer in the vehicular coordinate system is $f^{b}={\left[f_{x}^{b}\;f_{y}^{b}\;f_{z}^{b}\right]}^{T}$, and its projection in the navigation coordinate system is $g^{n}={\left[0\;0\;-g\right]}^{T}$. In the static case, there is the specific force equation ${\left(C_{b}^{n}\right)}^{T}{\vec{g}}^{n}+{\vec{f}}^{b}=\vec{0}$, which is ${\vec{f}}^{b}=-{\left(C_{n}^{b}\right)}^{T}{\vec{g}}^{n}$, expanded into the following component form.

(53) $$\left[\begin{array}{c} f_{x}^{b}\\ f_{y}^{b}\\ f_{z}^{b} \end{array}\right]=-{\left[\begin{array}{ccc} C_{11} & C_{12} & C_{13}\\ C_{21} & C_{22} & C_{23}\\ C_{31} & C_{32} & C_{33} \end{array}\right]}^{T}\left[\begin{array}{c} 0\\ 0\\ -g \end{array}\right]=\left[\begin{array}{c} C_{31}g\\ C_{32}g\\ C_{33}g \end{array}\right]$$

By solving the above equation, it can be obtained:

(54) $$\left[\begin{array}{c} C_{31}\\ C_{32}\\ C_{33} \end{array}\right]=\left[\begin{array}{c} f_{x}^{b}/g\\ f_{y}^{b}/g\\ f_{z}^{b}/g \end{array}\right]$$

The equations for the pitch angle θ and the cross-roll angle γ can be obtained by combining Equation (20) as [23]:

(55) $$\{\begin{array}{c} \theta =\arcsin(f_{y}^{b}/g)\\ \gamma =-\arctan(f_{x}^{b}/f_{z}^{b}) \end{array}$$

The magnetometer is solidly connected to the vehicular, and its coordinate system is the vehicular coordinate system b. The output of the magnetometer in the b system is $M^{b}={\left[\begin{array}{ccc} m_{x}^{b} & m_{y}^{b} & m_{z}^{b} \end{array}\right]}^{T}$. When the vehicular coordinate system b coincides with the magnetic geographic coordinate system $M^{m}={\left[\begin{array}{ccc} 0 & m_{N} & m_{U} \end{array}\right]}^{T}$, the magnitude of the magnetic field measured by the magnetometer at this time is $M^{b}={C_{n}^{b}|}_{\psi =\psi_{m}}M^{n}$. According to the formula and Equation (20), the following can be obtained:

(56) $$\left[\begin{array}{c} m_{x}^{b}\\ m_{y}^{b}\\ m_{z}^{b} \end{array}\right]=\left[\begin{array}{c} \cos\theta \sin\psi_{m}m_{N}+\left(\sin\gamma \cos\psi_{m}-\sin\theta \cos\gamma \sin\psi_{m}\right)m_{U}\\ \cos\theta \cos\psi_{m}m_{N}-\left(\sin\gamma \sin\psi_{m}+\sin\theta \cos\gamma \cos\psi_{m}\right)m_{U}\\ \sin\theta m_{N}+\cos\gamma \cos\theta m_{U} \end{array}\right]$$

Further, the heading angle can be obtained as:

(57) $$\psi =\psi_{m}+\mu =\arctan(\frac{m_{x}^{b}\cos\gamma +m_{z}^{b}\sin\gamma }{m_{y}^{b}\cos\theta +m_{x}^{b}\sin\gamma \sin\theta -m_{z}^{b}\cos\gamma \sin\theta })+\mu$$

(2) Alignment algorithm of initial heading angle and pitch angle based on dual-antenna GNSS

GNSS has the global positioning function, which can realize the all-weather accurate position navigation function. The positioning data provided by GNSS is also the main basis for the initial position alignment of the system. Dual-antenna GNSS can assist in determining the heading angle and pitch angle. In this paper, dual-antenna GNSS is mainly used to measure the heading angle and pitch angle when the satellite signal is excellent.

Dual-antenna GNSS has two satellite signal receiving antennas, the main antenna ANT1 and the secondary antenna ANT2, which are mounted on the longitudinal axis of the aircraft, and the baseline distance between the two antennas is D.

First, obtain the position coordinates $P_{ANT1}={\left[\begin{array}{ccc} \lambda_{1} & L_{1} & h_{1} \end{array}\right]}^{T}$ and $P_{ANT2}={\left[\begin{array}{ccc} \lambda_{2} & L_{2} & h_{2} \end{array}\right]}^{T}$ of ANT1 and ANT2 antennas, respectively, then subtract them to obtain the difference of position coordinates $\Delta E={\left[\begin{array}{ccc} \Delta e & \Delta n & \Delta u \end{array}\right]}^{T}$ of the two antennas, where Δe, Δn, Δu is the difference of the distance between the two antennas in the direction of east, north and sky, respectively, then substitute the above values into the following formula to obtain the heading angle ψ and pitch angle θ.

(58) $$\{\begin{array}{c} \psi =\arctan\left(\Delta e/\Delta n\right)\\ \theta =\arctan\left(\Delta u/\sqrt{\Delta e^{2}+\Delta n^{2}}\right) \end{array}$$

At the time of antenna installation, the distance D between the two antennas can be measured exactly, and, at the same time, based on the position coordinates provided by the two antennas, the calculated positioning distance $\tilde{D}=\sqrt{{\left(L_{1}-L_{2}\right)}^{2}+{\left(\lambda_{1}-\lambda_{2}\right)}^{2}+{\left(h_{1}-h_{2}\right)}^{2}}$ of the antennas can be obtained. By calculating the difference between D and $\tilde{D}$, the accuracy of the satellite positioning signal can be judged, and thus its credibility. The coordinates of the midpoint of the line connecting the two antennas are used as the vehicular coordinates of the aircraft so that they are used as the data for the initial alignment of the position.

### 4.5. Aircraft Flight State Recognition and Attitude Compensation

In different terrain environments, the navigable aircraft will face various environmental disturbances, and there may also be a satellite signal failure; when the integrated navigation system loses the update of the satellite measurement signal, the attitude angle may gradually drift, resulting in a gradual increase in the error angle, affecting the attitude accuracy of the navigation system. For this reason, an aircraft flight state identification and compensation procedure is added to use the IMU raw data for flight state identification in the case of satellite signal failure and to use the transverse roll and pitch obtained by accelerometer and the heading angle obtained by magnetometer for the quantitative update in the low maneuvering flight state to improve the attitude accuracy of the system and ensure the navigation safety of the aircraft [24].

**Figure 3:**
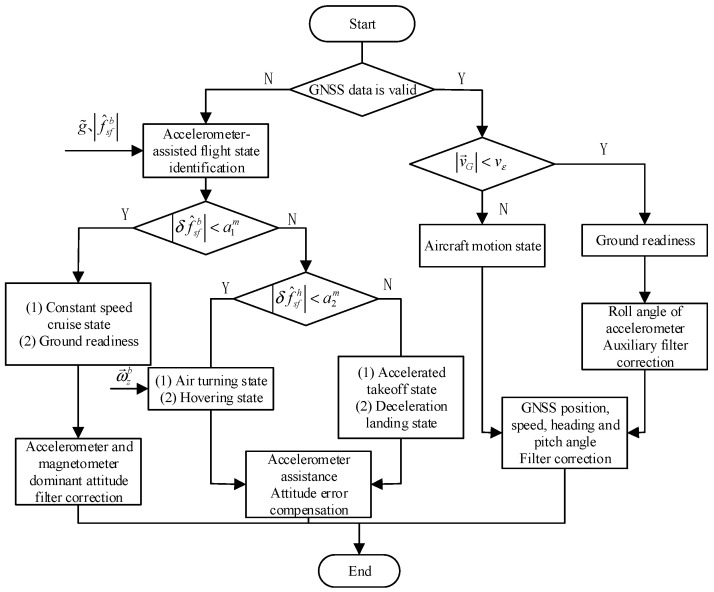
Schematic diagram of flight status identification and navigation correction data selection process.

This is shown in Figure 3 below, which shows a flowchart of the aircraft’s flight state identification and navigation correction data selection method by the navigation system.

In the static alignment phase of the through aircraft, the local reference gravitational acceleration $\tilde{g}$ is found using Equations (59) and (60).

(59) $${\left|f_{sf}^{b}\right|}_{k}=\sqrt{{\left(f_{sfx}^{b}\right)}^{2}+{\left(f_{sfy}^{b}\right)}^{2}+{\left(f_{sfz}^{b}\right)}^{2}}$$

(60) $$\tilde{g}=\left|{\tilde{f}}_{sf}^{b}\right|=\frac{{\left|f_{sf}^{b}\right|}_{1}+{\left|f_{sf}^{b}\right|}_{2}+\cdots {\left|f_{sf}^{b}\right|}_{n}}{n},\left(n=3000\right)$$

As GNSS system updates at 1 Hz, the data measurement update frequency of the system is 1 Hz. When the data sent by GNSS are received, they are parsed to judge the validity of the content, and, if the GNSS data are valid and available, they are used as the basis for judging the flight status of the aircraft according to the velocity of satellite decoding.

When $\left|{\vec{v}}_{G}\right|<v_{\varepsilon }$ ($v_{\varepsilon }$ is the velocity threshold parameter, the value is taken based on the velocity measurement noise of GNSS), the aircraft is judged to be in the ground-ready state. At this time, the heading, pitch, velocity and position information provided by GNSS are used for filtering correction, and the accelerometer is used for correction of the cross-roll angle in integration with Equation (36). When $\left|{\vec{v}}_{G}\right|\geq v_{\varepsilon }$, the aircraft is judged to be in the motion state, and, at this time, the filtering correction is made entirely using the heading, pitch, velocity and position information provided by GNSS, and no correction is made for the traverse angle.

When the GNSS data are invalid, the accelerometer is used for flight mode discrimination. According to Equation (60), the accelerometer output ${\left|{\hat{f}}_{sf}^{b}\right|}_{k}$ at moment k is obtained. When $\left|{\left|{\hat{f}}_{sf}^{b}\right|}_{k}-\tilde{g}\right|<a_{1}^{m}$ ($a_{1}^{m}$ is the threshold parameter; its value depends on the specific noise level setting of the environment where the navigation system is located), the aircraft is judged to be in steady state; at this time, the aircraft is in uniform cruise state or ground stationary state; this stage can use the accelerometer for attitude measurement correction.

(61) $${\left|{\hat{f}}_{sf}^{b}\right|}_{k}=\frac{{\left|f_{sf}^{b}\right|}_{k}+{\left|f_{sf}^{b}\right|}_{k-1}\cdots +{\left|f_{sf}^{b}\right|}_{k-m+1}}{m},\left(m=100\right)$$

When $\left|{\left|{\hat{f}}_{sf}^{b}\right|}_{k}-\tilde{g}\right|\geq a_{1}^{m}$, the output $\left|\delta {\hat{f}}_{sb}^{h}\right|$ of the horizontal biaxial accelerometer is calculated to determine the specific flight status, and the determination method is as follows.

(1) When $\left|\delta {\hat{f}}_{sb}^{h}\right|<a_{2}^{m}$ ($a_{2}^{m}$ is another threshold parameter based on the specific value of the horizontal accelerometer noise), integrated with the gyroscope data to determine, the aircraft may be in a turning state or circling state.
(2) When $\left|\delta {\hat{f}}_{sb}^{h}\right|\geq a_{2}^{m}$, the aircraft has a large maneuvering state and is in the takeoff or landing phase, and the accelerometer can be used to compensate for the error and reduce the error of attitude angle in this phase.

## 5. Experimental Verification and Analysis

To validate the actual performance as well as the reliability of this integrated navigation system, in this chapter, we detail an indoor static accuracy test of the equipment and UAV flight test to check the performance of the system under different environments and to verify the correctness of the system model and the feasibility of the system scheme.

### 5.1. Carry Out Indoor Static Experiment

**Figure 4:**
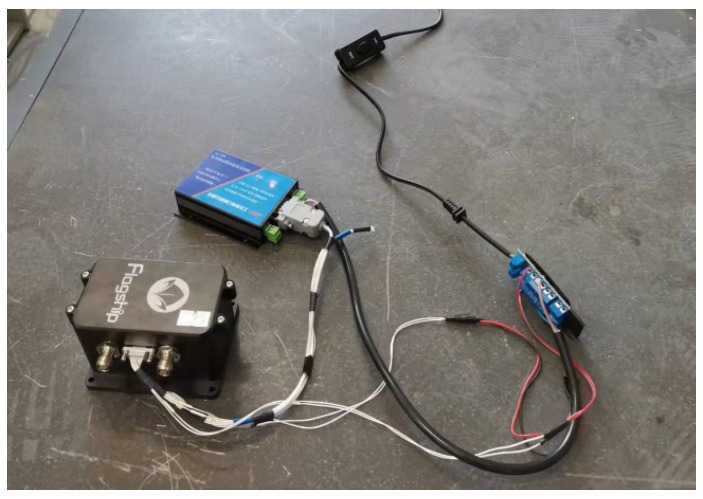
Field experiment diagram of static test.

The first test is to test the static performance parameters of the system in an indoor environment. The main components of the indoor static experiment are an integrated navigation device body and a data recorder. The device was placed on a marble table in the laboratory, the power was started and, after the initialization of the device was completed, data were sent to the serial data logger and 30 min data were collected. Figure 4 shows the field experiment for static testing.

**Figure 5:**
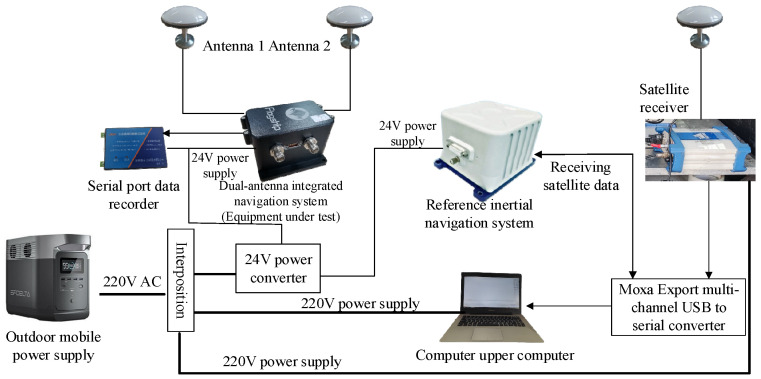
Installation diagram of experimental system.

The experimental system installation diagram is shown in Figure 5.

The entire system is powered by an outdoor mobile 220 V AC supply. The tested dual-antenna GNSS/MINS integrated navigation system, reference fiber inertial navigation system and serial port data recorder use 24 V DC power supply provided by an AC/DC power adapter, while the laptop and reference satellite receiver use 220 V AC power supply.

The tested integrated navigation system receives satellite data through two satellite antennas and sends the final navigation solution data to a serial port data recorder for storage. The distance between the two satellite antennas is 1.5 m. The reference inertial navigation device reads the satellite data sent by the satellite receiver through a Moxa Upload multi-channel serial port USB to serial port converter and performs an integrated navigation solution that then sends the navigation data through the converter to the uplink computer for saving.

**Figure 6:**
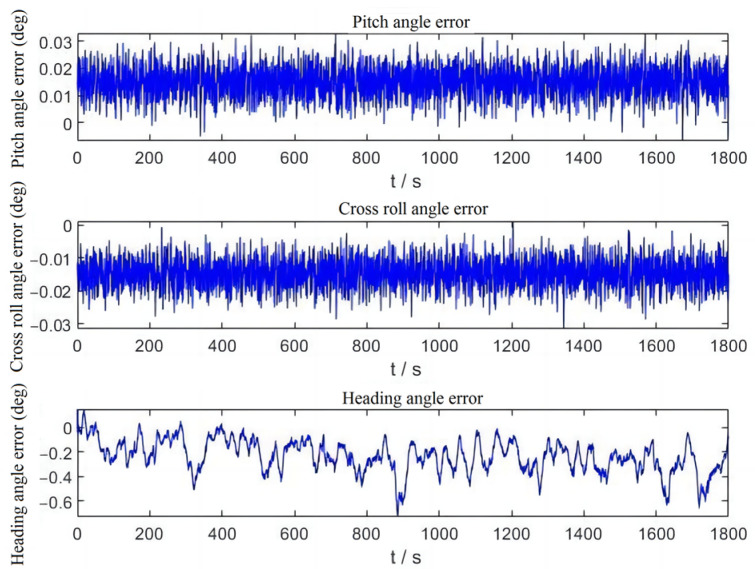
Static test attitude error output result graph.

The test results are shown in Figure 6.

In the indoor static test situation, the system first detects that the satellite signal is not available, so it performs flight status identification by accelerometer and determines that the current device is in ground-ready state. During the ground-ready state without satellite signal, the system will use an accelerometer to correct the traverse and pitch angles of the attitude and a magnetometer to correct the heading angle. From the results in Table 2, the errors of pitch and roll angles in static condition are extremely small, and the RMS is within 0.75°. The error of heading angle is larger than the pitch and roll angles corrected by accelerometer due to fusion correction by magnetometer, but the error RMS is also within 1°.

### 5.2. Conduct UAV Flight Test

This flight test will carry the system inside the aircraft near the propeller using the on-board 28 V DC to power the test equipment, using the on-board 12 V battery to power the industrial serial data logger, the reference equipment is the on-board main fiber optic inertial guidance and the GPS signal and heading are provided by the aircraft. Since the aircraft does not provide velocity information, the system will not perform velocity measurement update and comparison in this subsection. The flight process is divided into several phases, such as pre-takeoff preparation, takeoff, turn, cruise and landing, etc. This flight test will focus on analyzing the data of the first 2190 s, which contain the additional four phases of the flight process except for landing.

**Figure 7:**
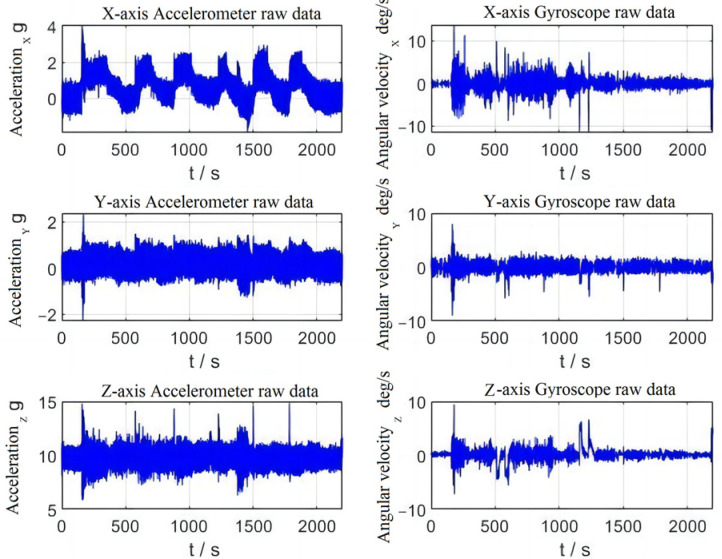
Flight test accelerometer and gyroscope data.

From Figure 7, it can be demonstrated that the aircraft will have an overall large amount of noise due to the vibration of the propeller. Even when the aircraft is in the ground preparation phase between 0 and 190 s, the gyroscope and accelerometer have a large amount of noise, but it can be considered as zero-mean noise, which has minor effect on later data solving. Compared with the sports car test, when the aircraft in the flight test undergoes dynamic changes, the amount of data change of its accelerometer and gyroscope is relatively small, the dynamic impact is smaller and the navigation effect can be expected to be better.

**Figure 8:**
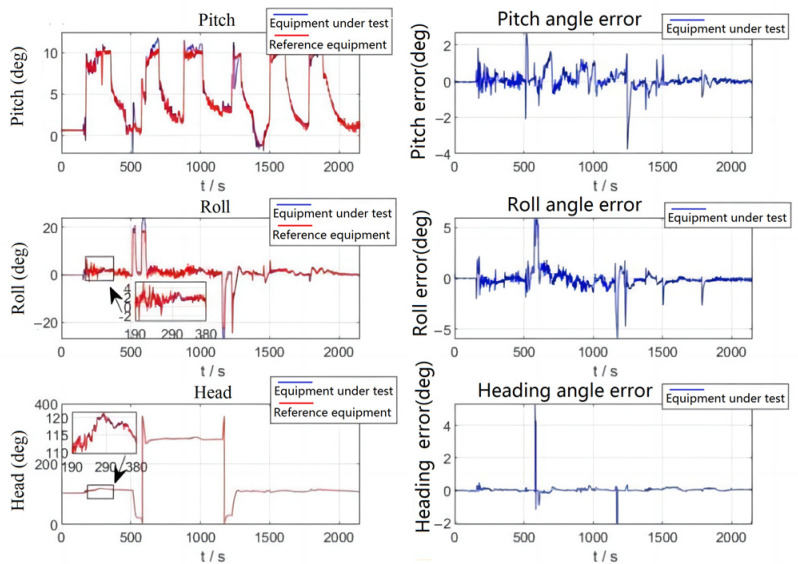
Flight test attitude comparison and attitude error.

As shown in Figure 8, the aircraft is in the ground preparation state during 0–190 s, and the errors of the three attitude angles are relatively small. During the period of 191–380 s, the aircraft is in the takeoff phase, at which point the aircraft raises its head and the error of the pitch angle increases but remains within the range of 2°. The error of the cross-roll angle also has some fluctuations and remains within 1.5 degrees overall, and the error of the heading angle is small. During the period of 500–610 s, the aircraft starts the first large-angle turn, which leads to an increase in error, and the error exceeds 2°. During 500–610 s, the aircraft starts to make the first large-angle turn because there is no correction for the cross-roll angle at this time, which leads to an increase in error and further a brief large error fluctuation in the pitch angle; the error was more than 2°; this error was quickly corrected. Heading angle due to focus on stability, resulting in a slightly slower following velocity, causes large error, about 4°; about 1–2 s later, the system followed on the true heading angle and the amount of error returned to normal. After that, the aircraft made five climbs and one large maneuvering turn, during which the attitude angle error would increase, but the maximum error was within 5° and the correction velocity was fast.

The following table shows the quantitative analysis of the flight test attitude angle errors. In Table 3, the heading angle error is the smallest and the cross-roll angle error is a bit larger than the pitch angle error, but the root mean square of the error is within the allowable range of the index.

**Figure 9:**
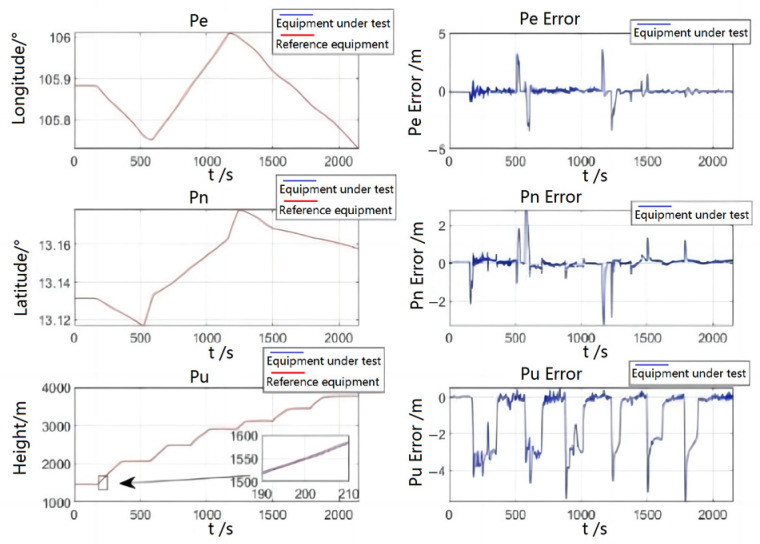
Flight test position comparison and error.

In Figure 9, the error in longitude and latitude of the system is relatively small, and a slightly larger amount of error will be generated during the takeoff, climb and turn phases. Regarding altitude, because the system depends on the correction of the satellite, there is a certain delay in the satellite signal, which will produce slightly larger error in the climb phase, about 4 m. Although the altitude error will be reduced if the measurement noise of the altitude is reduced at this time, the northward and skyward position error will increase, so a compromise is needed. When the aircraft is flying smoothly, the altitude error can be corrected quickly.

The following table shows the quantitative analysis of the position error of the flight test. From Table 4, the root MSE of the position in the east direction and north direction are around 0.45 m, and the root MSE in the sky direction is larger, which can also meet the requirement of less than 1.5 m.

**Figure 10:**
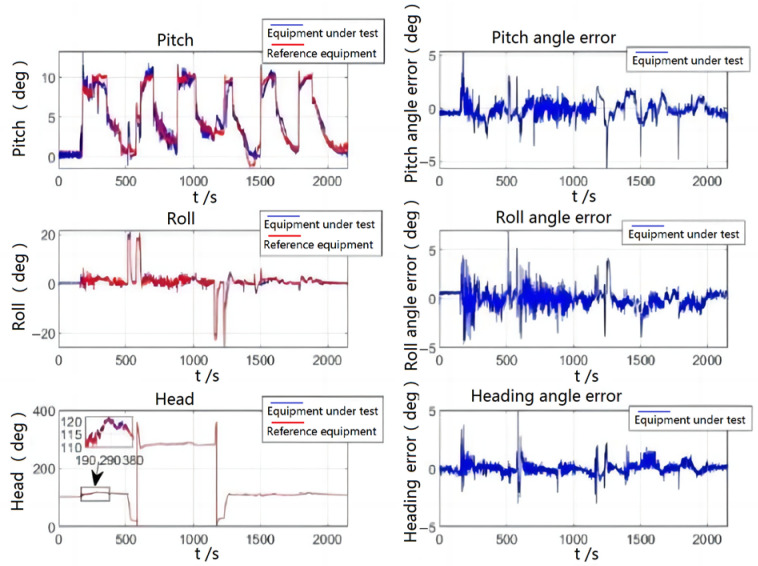
Inertial guidance attitude error.

The system’s attitude solving effect in the absence of satellite is verified. After the 30S initial alignment of the system, the GNSS signal of the system is disconnected so that the system only uses the raw IMU data for attitude and heading angle solution and filtering, and the experimental effect is shown in Figure 10. When there is no GNSS signal, the system will use IMU data for flight attitude discrimination and then use accelerometer for attitude angle correction in the low acceleration phase of flight. From Figure 8, the attitude angle solution using accelerometer will have a larger amount of error compared with the attitude acquired by GNSS, but the systematic error RMS is guaranteed to be within 1 degree, which can be used as an inertial guide to provide higher accuracy attitude navigation information when there is no satellite signal from the aircraft.

Quantitative analysis of the attitude error is shown in Table 5. Through flight test verification, the effectiveness of the algorithm designed in this paper, using the angle, velocity and position information provided by GNSS and MEMS inertial guidance system for high precision data fusion, when the satellite signal fails, the IMU data alone can also be used to obtain better attitude solution accuracy to ensure the flight safety of the aircraft.

## 6. Conclusions

By studying the discrete Kalman filter algorithm, the state-space model of the system is established based on the system error equation in this study. The mathematical model of the integrated dual-antenna GNSS/MINS navigation system is established and the integrated inertial guidance algorithm of Jet-Link is designed. The system is also tested in indoor static tests and UAV flight experiments to verify the attitude performance of the system, especially when it meets the index requirements without satellite signals. The experiments verified that the attitude, velocity and position accuracy through the system during take-off, climbing and turning are elevated and the errors are within the index range. It is realized that the attitude accuracy and position accuracy of the system meet the system index requirements when a GNSS signal is available. When there is no GNSS signal correction, the accelerometer can also be used to obtain a better attitude solution to ensure safe return and landing of the aircraft under GNSS signal rejection environment.

Future research will investigate the method to maintain the accuracy of MEMS inertial guidance solution for a longer duration in the absence of a satellite signal. Combined with the current situation of poor satellite signal due to complex terrain and mountainous plateaus in China, an airborne tight integration navigation algorithm will be studied. Using the existing hardware equipment, a more efficient and feasible algorithm will be explored to realize an algorithm solution of superior accuracy using low-cost computing units.

## Figures and Tables

**Table 1:** Main index requirements for an integrated navigation system.

Entry Name		Indicator Parameters
Heading Accuracy	Double GNSS (2 m Baseline)	1° (RMS)
	Heading holding (GNSS failure)	1.5 °/min (RMS)
Attitude accuracy	GNSS valid (single point L1/L2)	0.75° (RMS)
Horizontal positioning accuracy	GNSS valid, single point L1/L2	1.5 m (RMS)
Horizontal velocity accuracy	GNSS valid, single point L1/L2	0.2 m/s (RMS)
Gyroscope	Measuring range	±450 °/s
	Bias stability	6 °/h
Accelerometer	Measuring range	±16 g
	Bias stability	0.3 mg
Environmental indicators	Working temperature	−40 °C~+60 °C
	Vibration	5~2000 Hz, 2 g
	To attack	30 g, 11 ms

**Table 2:** Static test attitude angle error.

Parameter	Pitch Angle Error (°)	Cross Roll Angle Error (°)	Heading Angle Error (°)
Means	0.01493	−0.01472	−0.2335
Maximum value	0.0329	0.001092	0.1458
Minimum value	−0.00667	−0.03147	−0.7275
RMS	0.004792	0.003721	0.1307

**Table 3:** Flight test attitude error.

Parameter	Pitch Angle Error (°)	Cross Roll Angle Error (°)	Heading Angle Error (°)
Means	0.0522	−0.1148	0.02089
Maximum value	3.01	5.771	4.312
Minimum value	−2.784	−5.935	−2.123
RMS	0.4574	0.7051	0.2251

**Table 4:** Flight test position error.

Parameter	Eastward Position Error (m)	Northward Position Error (m)	Skyward Position Error (m)
Means	0.03407	0.0341	−1.035
Maximum value	3.601	2.821	0.5304
Minimum value	−3.467	−3.299	−5.723
RMS	0.4995	0.4323	1.445

**Table 5:** Inertial guidance attitude error.

Parameter	Pitch Angle Error (°)	Cross Roll Angle Error (°)	Heading Angle Error (°)
Means	−0.139	−0.00824	0.03385
Maximum value	5.352	6.955	4.987
Minimum value	−5.717	−4.633	−2.936
RMS	0.8448	0.9444	0.4876

## Data Availability

Not applicable.
